# Assessing the restorative environment of pocket parks in old urban districts of Chinese cities in the context of healthy aging

**DOI:** 10.3389/fpubh.2026.1861979

**Published:** 2026-07-15

**Authors:** Di Hu, Yi Lu, Yuhan Guo, Yifan Wu, Guoqing Shen, Lanxi Ma, Lei Li

**Affiliations:** 1 Academy of Arts and Design, Anhui University of Technology, Maanshan, China; 2 School of Agriculture and Biology, Shanghai Jiao Tong University, Shanghai, China; 3 School of Arts, Nanjing University of Information Science and Technology, Nanjing, China

**Keywords:** age-friendly environments, healthy aging, old urban districts, older adults, pocket parks, restorative environments

## Abstract

**Background:**

In the context of healthy aging, pocket parks in old urban districts function as small-scale green open spaces that are easily accessible in older adults’ daily lives. However, their restorative environmental characteristics and appropriate assessment methods remain insufficiently examined.

**Methods:**

This study focused on three representative pocket parks in the old urban districts of Maanshan, China, and developed an evaluation framework tailored to older adults’ everyday use contexts under the theoretical lens of restorative environments. An integrated approach combining the Fuzzy Delphi Method, the Analytic Hierarchy Process, and Fuzzy Comprehensive Evaluation was applied.

**Results:**

The evaluation system comprised five dimensions and 25 indicators. Among the five dimensions, safety received the highest weight, followed by comfort and mobility, whereas richness and attractiveness had lower weights. The three pocket parks showed different levels of restorative-supportive performance, with Zhenqu Pocket Park obtaining the highest comprehensive score, followed by Kangle Pocket Park and Zhumeng Pocket Park. Weight sensitivity analysis showed that this ranking remained stable across all perturbation scenarios. In addition, the indicator-level non-parametric comparison confirmed significant differences in the score profiles of the three parks. Despite these differences, all three parks shared a similar structural pattern, characterized by relatively strong safety performance but insufficient richness and attractiveness.

**Conclusion:**

The findings indicate that improving the restorative quality of pocket parks in old urban districts should not rely solely on increasing greenery or facilities, but should instead emphasize the coordinated enhancement of safety, walkability, comfort, richness, and experiential attractiveness. This study provides empirical evidence for restorative environment assessment and practical support for the development of age-friendly pocket parks.

## 1 Introduction

Population aging has emerged as one of the most consequential demographic and social transformations of the twenty-first century. United Nations projections indicate that, by 2050, the proportion of the global population aged 65 years and above will rise from approximately 10% in 2022 to 16%, meaning that one in every six people worldwide will be an older adult (1). Against this backdrop, the World Health Organization (WHO) advanced the concept of healthy aging, defining it as the process of developing and maintaining the functional ability that enables well-being in later life, thereby shifting the policy focus beyond longevity alone toward the preservation of everyday functioning and quality of life among older adults (2). In response, the United Nations officially launched the Decade of Healthy Aging (2021–2030), which identifies the creation of age-friendly environments and the advancement of age-friendly communities and cities as central priorities for coordinated global action (3, 4). In China, the population aged 60 years and older is expanding rapidly and is projected to exceed 400 million by 2035. At the national level, strategic frameworks such as the active response to population aging, Healthy China 2030, and healthy aging policies have begun to mobilize action across health and care services, age-friendly environmental modification, and community-based support systems (5). At the community scale, healthy aging depends not only on medical and care services, but also on nearby public spaces that support older adults’ everyday activities, neighborhood interaction, and psychological adjustment. In this context, pocket parks generally refer to small-scale public green spaces embedded within the built urban environment. They are typically characterized by limited site area, proximity to residential neighborhoods, strong walkable accessibility, and frequent everyday use. Their spatial composition, organization of greenery, opportunities for staying, and conditions for activity can influence users’ resting, social interaction, and health-promoting use. As an important form of small community green space, pocket parks can provide older adults with frequently used places for walking, resting, and social engagement. Meanwhile, pocket parks also have an intergenerational sharing function, as they can support children’s activities, family companionship, neighborhood interaction, and everyday public life within the community. However, in old urban districts with a high degree of population aging, older adults are often among the most stable and frequent user groups of these spaces. Within this policy and demographic context, a critical public health question is how improvements to pocket parks, public green spaces, and other everyday accessible open spaces can better support healthy aging. This issue has become a shared priority across international agendas promoted by the United Nations and WHO, as well as within China’s domestic policy framework.

In China, routine walking, neighborhood strolling, and park visitation constitute some of the most common forms of daily physical activity and social engagement among older adults. Existing evidence indicates that urban parks can substantially enhance physical activity, social interaction, and self-rated health in later life (6, 7). As people age, their activity spaces tend to contract; compared with large comprehensive parks located farther from home, older adults rely more heavily on small-scale open spaces that are accessible on foot within their immediate neighborhoods (8). Against this backdrop, Chinese cities have increasingly incorporated pocket parks and other small green spaces into the development of the “15-min living circle” positioning them as an important planning instrument for improving community livability and strengthening local public service provision (8–10). Constrained by fiscal capacity, limited land reserves, and development intensity, newly developed urban districts generally benefit from more abundant, newer, and better-integrated green space and public service infrastructure, whereas older urban districts are more likely to experience deeper population aging, slower spatial renewal, insufficient provision of public space and greenery, and uneven environmental quality (11). In this context, embedding pocket parks into residual parcels, vacant lots, and underutilized land within old urban districts offers not only a cost-effective strategy for improving older adults’ everyday activity environments and psychological well-being, but also an approach that has already been widely adopted and validated in urban regeneration practice across China (12, 13). Yet, within the broader agenda of healthy aging, systematic evidence remains limited as to whether pocket parks in older urban districts possess restorative environmental qualities and, more importantly, how such qualities can be rigorously evaluated.

Within the fields of healthy aging and restorative environment research, restorative environment theory provides a critical conceptual lens for understanding how natural settings promote health in later life. Two theoretical frameworks have been especially influential in this literature. Attention Restoration Theory (ART) explains how certain environments facilitate the recovery of depleted attentional resources and cognitive functioning, whereas Stress Recovery Theory (SRT) emphasizes the capacity of natural environments to mitigate physiological stress and negative affect. Taken together, these frameworks delineate complementary pathways through which nature supports psychological and physical restoration. More specifically, ART posits that prolonged engagement in tasks requiring directed attention leads to attentional fatigue, while environmental qualities such as soft fascination, being away, extent, and compatibility can restore attention and cognitive functioning by engaging involuntary attention (14, 15). From a psychophysiological perspective, SRT further suggests that exposure to natural landscapes containing elements such as vegetation and water can reduce sympathetic arousal and alleviate tension and anxiety (16, 17). The classic study by Ulrich et al. on hospital window views further suggests that exposure to natural scenery may be associated with stress reduction and restorative processes (18). Building on these theoretical advances, Hartig et al. developed the Perceived Restorativeness Scale (PRS), which has since been widely used to assess the restorative potential of different types of green space (19). A growing body of empirical research has shown that higher levels of perceived restorativeness in urban parks, commonly measured using the PRS, are generally associated with more positive emotional experiences as well as greater psychological well-being and perceived health benefits (19, 20). In recent years, small urban green spaces and pocket parks have also entered the scope of restorative environment research (13, 20–22). In addition, a systematic review by Ohly et al. confirmed the restorative effects of natural environments on attentional recovery (23). Taken together, the stress-alleviation and attentional-recovery mechanisms articulated by ART and SRT, together with the substantial empirical evidence generated through PRS-based research, provide a robust theoretical and evidentiary foundation for evaluating the health value of urban pocket parks from a restorative environment perspective, particularly with respect to their potential benefits for older adults.

Methodologically, existing studies on the evaluation of parks and age-friendly public spaces have broadly converged on three technical approaches. The first relies on subjective perception-based methods, including questionnaire surveys, perceptual scales, and post-occupancy evaluation (POE), to assess age-friendliness and health-related outcomes from the perspective of older adults’ lived experience and use of space (24). The second adopts objective indicator-based approaches, drawing on measures such as green-space accessibility, vegetation cover, and facility adequacy, and commonly integrates GIS and spatial analysis to construct evaluative frameworks for environmental quality and spatial equity (25). The third introduces multi-criteria decision-making methods to assign weights across multidimensional indicators and to generate composite evaluations under conditions of complexity and uncertainty; among these, the AHP, FCE, and hybrid AHP–FCE models have been applied to the assessment of public spaces in old urban districts, park environments, and pocket parks (26–28). Within the context of healthy aging, emerging studies have begun to examine the restorative potential of public open spaces in older urban neighborhoods, highlighting the pivotal roles of safety, comfort, and naturalness in shaping restorative experiences among older adults (29). Overall, however, the literature has concentrated primarily on large urban parks or community environments at a broader scale. Comprehensive evaluation of the restorative qualities of pocket parks in old urban districts remains comparatively underdeveloped, particularly studies capable of integrating multidimensional indicators, expert judgment, and older adults’ subjective perceptions within a unified assessment framework.

In this study, “restorative environmental quality” refers to the capacity of pocket parks to provide environmental conditions that may facilitate restorative experiences among older adults. It does not directly denote psychological or physiological restoration outcomes that have already occurred, nor is it identical to perceived restorativeness as measured by the PRS. Rather, the restorative support potential examined in this study refers to the extent to which pocket parks provide conditions related to accessibility and movement, perceived safety, physical comfort, environmental richness, and perceived attractiveness. These conditions are understood as potentially supporting attention restoration, stress reduction, emotional relaxation, everyday activities, and social interaction among older adults.

Based on this conceptual positioning, the present study takes pocket parks in old urban districts of Chinese cities as its research object. Under the combined framework of healthy aging and restorative environment theory, it develops an evaluation framework for restorative-supportive environments oriented toward older adults’ everyday use contexts and empirically evaluates three representative pocket parks in the old urban districts of Maanshan. Specifically, this study addresses the following three questions:

1. Which core dimensions and indicators constitute the restorative-supportive environmental quality of pocket parks in old urban districts?
2. Based on expert judgment, how are these dimensions and indicators prioritized, and what evaluation priorities are reflected in their weighting structure?
3. What performance characteristics do the three representative pocket parks exhibit in terms of restorative-supportive environmental quality, and in which domains are their respective strengths and weaknesses manifested?

By answering these questions, this study aims to clarify the evaluation dimensions, indicator weights, and case-specific performance characteristics of restorative-supportive environmental quality in pocket parks in old urban districts. In doing so, it provides methodological guidance and empirical evidence for community-level micro-renewal and the development of age-friendly pocket parks under the agenda of healthy aging.

## 2 Literature review

### 2.1 Restorative environment research oriented toward healthy aging

The concept of healthy aging emphasizes the maintenance of functional ability across the life course. Within this framework, the physical environment and access to everyday outdoor spaces are increasingly recognized as critical determinants of health and well-being in later life (30, 31). In environmental psychology and health geography, restorative environments are commonly invoked to explain how natural or semi-natural settings facilitate stress reduction and attentional recovery. This body of research is primarily grounded in two influential theoretical frameworks: ART and SRT. ART posits that environmental qualities such as being away, fascination, extent, and compatibility promote the recovery of directed attention, thereby alleviating cognitive fatigue and improving emotional states. By contrast, SRT highlights the capacity of relatively non-threatening natural settings to support psychophysiological restoration by reducing stress and negative affect. Classic studies have further suggested that visual exposure to natural landscapes may play a meaningful role in restorative processes (17, 18). Taken together, a restorative environment is not merely a space that contains natural elements. Rather, it refers to an environment that can support restorative processes through low-effort perception, environmental attraction, emotional relaxation, and compatibility with users’ needs and activities. When this theoretical understanding is placed within the context of healthy aging, pocket parks show particular contextual relevance. Because they are small in scale, close to residential neighborhoods, and accessible on foot, pocket parks are often embedded in older adults’ everyday activity routes. For older adults, these spaces provide not only opportunities for contact with nature, but also settings for walking, short stays, light physical activity, and neighborhood interaction.

Accordingly, when pocket parks offer safe movement, comfortable conditions for staying, moderate environmental variation, and opportunities for low-disturbance social interaction, the mechanisms emphasized by ART and SRT—attention restoration, emotional relaxation, and stress reduction—may be incorporated into older adults’ everyday use processes. From the perspective of healthy aging, it is therefore both theoretically justified and practically significant to conceptualize pocket parks as small-scale green spaces that are reachable, usable, and socially supportive in older adults’ daily lives, and to further evaluate their restorative environmental characteristics and health-supportive value.

### 2.2 Assessment scales and evaluation methods for restorative environments

Methodologically, restorative environment research has evolved along three complementary lines of inquiry. The first is a subjective measurement tradition centered on psychometric scales. Representative instruments include the PRS developed by Hartig et al. to assess the restorative qualities of a setting (19), and the Restoration Outcome Scale proposed by Korpela et al. to capture individuals’ restorative outcomes after staying in a given environment (32). The second consists of systematic syntheses and integrative evaluations of ART-based research and attentional restoration processes. Existing reviews suggest an overall positive association between exposure to natural environments and attention restoration outcomes (33). However, substantial heterogeneity remains in task paradigms and in the operationalization of attentional processes, underscoring the need for more clearly specified measurement frameworks and more comparable study designs. The third is multi-criteria comprehensive evaluation for planning and design decision-making. Within this approach, the Analytic Hierarchy Process is used to hierarchically structure evaluation criteria and derive their relative weights (34). At the same time, fuzzy set theory and FCE are introduced to characterize the inherent vagueness and uncertainty involved when respondents move from perceiving site characteristics, to reporting psycho-emotional and physical experience, and ultimately to expressing judgments through linguistic categories such as “very good,” “average,” or “poor” (35). In addition, the Fuzzy Delphi Method can be employed at the indicator-screening stage to consolidate expert judgment and build consensus. Originally proposed by Ishikawa et al., this method extends the traditional Delphi technique by explicitly accommodating fuzziness and uncertainty in expert opinion (36). Since then, related methods have been applied to ecological environmental quality assessment, walkability evaluation, urban regeneration, and studies of health-related quality in public space (37, 38).

In China, the research focus has gradually shifted from the macro-level quantity of green space toward the micro-level quality of spatial experience, with growing attention to the mechanisms underlying restorative effects and to systematic indicator-based evaluation. Micro-scale environments are especially important for older adults. As people age, their activity spaces tend to contract, while their walking capacity and willingness to make longer-distance trips often decline. As a result, older adults’ everyday outdoor activities depend more heavily on small-scale open spaces near their residences that are accessible on foot and capable of supporting short-term stays (8). Community green spaces, pocket parks, and public spaces around residential neighborhoods should therefore be understood not merely as supplementary green resources, but as important environmental carriers that support walking, resting, neighborhood interaction, and psychological restoration in later life. This also means that restorative environment assessment for older adults should move beyond the total quantity of green space or macro-scale accessibility, and should instead give greater attention to micro-scale conditions such as route continuity, safety, seating, shading, facilities, and perceived experience. From the perspectives of public health and urban regeneration within the existing built environment, prior studies have developed indicator systems for green open spaces in residential communities located in old urban districts and have used multi-criteria decision-making methods to propose renewal strategies better aligned with resource-constrained, precision-oriented interventions (39). At the level of spatial provision, Wei et al. examined the renewal of IGS in older residential neighborhoods and public perceptions of such spaces, thereby offering practical insights for the governance of small-scale green spaces such as pocket parks (40). More robust experimental evidence on restorative mechanisms in public spaces of old urban districts has also begun to emerge. Using typical old residential neighborhoods in Tianjin as a case, Zhou et al. combined virtual reality, subjective evaluation, and attention testing to reveal the pathways through which visual and acoustic landscape factors jointly shape attention restoration (41).

Overall, existing studies have largely focused on single communities or individual parks, with relatively limited attention paid to clusters of pocket parks within old urban districts. Comprehensive restorative assessments conducted across multiple pocket parks therefore remain scarce. Accordingly, evaluating the restorative qualities of pocket park networks in representative cities would help address current gaps in relation to spatial scale, park typology, and age-friendliness, while also generating evidence to inform health-oriented optimization of existing urban spaces in old districts.

### 2.3 Current state of restorative assessment for pocket parks and age-friendly public spaces

As a form of urban green space characterized by its small scale, embedded location, and everyday neighborhood accessibility, the restorative potential of pocket parks has increasingly attracted international scholarly attention. In their evaluation of visual stimuli in parks, Nordh and Østby demonstrated that the spatial composition and design features of small parks significantly shape individuals’ subjective judgments of restfulness and restoration, thereby providing early empirical support for the assessment of restorative qualities in pocket parks (42). Focusing on health-promoting patterns of use in pocket parks, Peschardt and Stigsdotter further showed that these spaces are frequently used for resting and socializing, and that their health-related benefits are associated with such attributes as opportunities for lingering, degree of disturbance, spatial enclosure, and the organization of greenery within the site (43, 44). More recent empirical work has moved toward direct comparison of restorative outcomes across park types. For example, one study comparing psychological and physiological restoration in pocket parks and community parks found that, when design quality is high and the use context is well matched to users’ needs, pocket parks can generate restorative effects comparable to those of community parks. Importantly, these effects appear to be shaped more strongly by site quality and use context than by park size alone (45). At the same time, experimental and modeling studies have begun to examine the mechanisms through which specific environmental features of pocket parks influence psychological restoration, with findings indicating that vegetation structure and activity facilities can affect both the alleviation of mental fatigue and the quality of restorative experience (22, 46).

Nevertheless, important gaps remain in research on pocket parks located in old urban districts under the framework of healthy aging. First, although the existing evidence broadly supports the restorative benefits of contact with nature, systematic reviews have shown that studies differ substantially in how attentional recovery is conceptualized, which measurement tools are used, and how experimental contexts are controlled (23). As a result, the strength and comparability of evidence linking nature exposure to restorative outcomes still need to be strengthened in real-world settings that more closely approximate everyday life (33). Second, older adults are more sensitive to environmental risk, as well as to issues of accessibility and facility provision. Needs related to safety, comfort, accessibility, opportunities to stay, and opportunities for social interaction shape both patterns of use and subjective experience, and these factors likely interact with restorative processes. For instance, research evaluating the restorative qualities of public spaces in old urban districts has shown that safety may emerge as a highly weighted dimension. This suggests that the restorative value of pocket parks cannot be adequately represented by the amount of greenery alone; rather, assessment should incorporate a broader set of multidimensional environmental quality indicators (29). Third, the evaluation of age-friendly public spaces is increasingly moving toward multi-indicator integrated decision-making oriented to the needs of older adults. Yet the existing evidence remains concentrated on urban community parks or general public spaces. Indicator adaptation for pocket parks in old urban districts remains insufficient, evidence on weighting structures is still limited, and empirical applications of comprehensive evaluation remain relatively scarce.

From the perspective of healthy aging, these research gaps are not limited to the methodological evaluation of pocket parks. They also concern whether everyday environments in old urban districts can support older adults’ functional ability. As micro-scale green open spaces that are accessible in daily life, pocket parks may contribute to the maintenance of older adults’ quality of life by supporting walking, resting, social interaction, emotional adjustment, and light physical activity. In this sense, evaluating the restorative-supportive environmental quality of pocket parks in old urban districts is essentially a way to identify the environmental support conditions required for healthy aging. Against this background, the present study focuses on pocket parks in old urban districts and develops a restorative evaluation framework tailored to older adults’ everyday use contexts. It further applies an integrated FDM–AHP–FCE approach to conduct a comprehensive assessment, thereby addressing the above gaps in spatial scale, indicator adaptation, weighting structure, and empirical application.

## 3 Methods

### 3.1 Study area and case selection

Maanshan, Anhui Province, is a typical old industrial city in China. Its old urban districts contain many residential communities historically developed around mining and industrial enterprises, resulting in compact spatial structures and a relative shortage of public green and everyday open spaces. The city also faces a high level of population aging. In 2022, residents aged 60 years and above numbered 486,000, accounting for 22.24% of the total population (47). These demographic and spatial conditions make Maanshan’s old urban districts a relevant setting for examining how small-scale green open spaces support older adults’ everyday outdoor activities.

To address the shortage of accessible open spaces, Maanshan has promoted the “Ma Xiaobai” pocket park system by reusing residual land, vacant parcels, and spaces near residential communities (12, 48). This initiative has gradually created a micro-scale green open-space network close to residential areas, providing nearby places for walking, resting, social interaction, and low-intensity physical activity. Within this system, pocket parks are not isolated green spaces, but everyday neighborhood environments embedded in residents’ daily mobility and activity routes.

**Figure 1:**
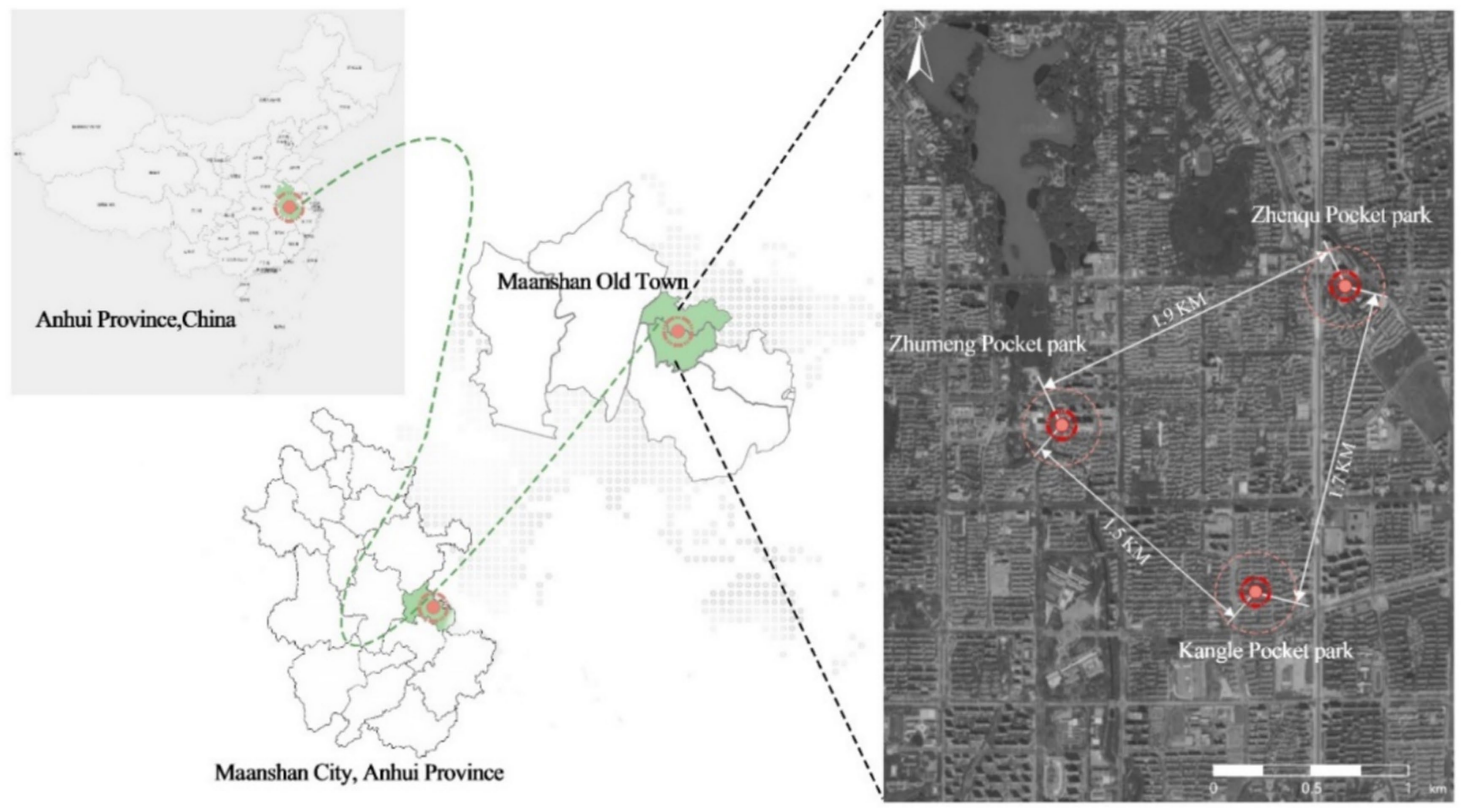
Location of the study area.

Based on this context, this study selected Kangle Pocket Park, Zhumeng Pocket Park, and Zhenqu Pocket Park as empirical cases (Figure 1). All three parks are located within residents’ everyday living circles, close to residential communities and daily mobility routes, and mainly serve nearby residents, especially older adults with frequent outdoor activity needs. These shared characteristics make them suitable for examining restorative-supportive environmental quality under the same urban regeneration context.

**Figure 2:**
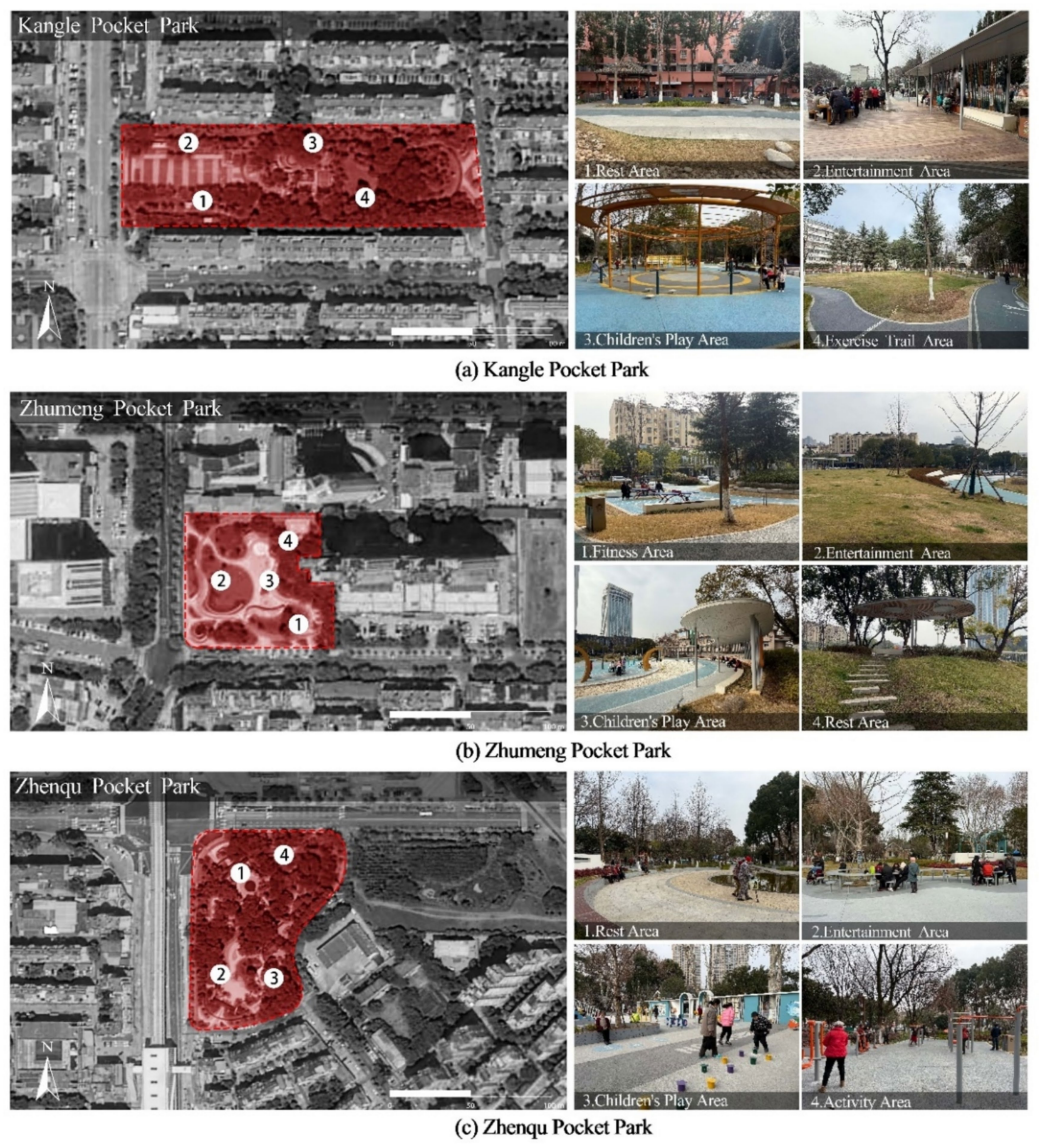
Spatial boundaries and on-site use contexts of the three case pocket parks: **(a)** Kangle Pocket Park; **(b)** Zhumeng Pocket Park; **(c)** Zhenqu Pocket Park.

The three parks also differ in spatial form, site area, facility configuration, and activity organization (Table 1). As shown in Figure 2, Kangle Pocket Park has a linear layout adjacent to residential areas and daily pedestrian routes, mainly supporting walking, resting, and neighborhood staying. Zhumeng Pocket Park uses a looped pathway to connect multiple activity zones. Zhenqu Pocket Park is relatively larger and integrates walking paths, pavilions and corridors, play facilities, and sports functions. The rest and activity areas marked in Figure 2 indicate spatial nodes frequently used by older adults during field observation and the questionnaire survey. These spaces are not exclusive to older adults and may also serve children, family members, and other community residents, reflecting a degree of intergenerational sharing.

**Table 1:** Basic characteristics and comparison of the three pocket park cases.

Site	Location and scale	Spatial form and organization	Main functions and use characteristics
Kangle pocket park	Located on the north side of Kangle Jiayuan; approximately 210 m from east to west and 60–80 m from north to south; total area about 13,700 m^2^	Linear space adjacent to residential areas and daily pedestrian routes	Mainly supports walking, resting, children’s activities, and everyday use; serves residents in surrounding communities
Zhumeng pocket park	Located at the northeastern corner of the intersection of Yanyang Road and Huafei Road; total area about 12,400 m^2^	Structured as “one loop and six zones,” with a looped pathway connecting multiple activity zones	Provides woodland leisure, active recreation, and fitness activity spaces; shows relatively strong activity participation
Zhenqu pocket park	Located in a community service-oriented pocket park area in an old urban district; total area about 16,450 m^2^	Relatively large spatial scale; includes walking paths, pavilions and corridors, play areas, and sports spaces	Combines resting, exercise, social interaction, and neighborhood service functions; shows relatively high everyday use intensity

Overall, the three selected parks provide a useful basis for comparative case analysis. They belong to the same municipal pocket park system and share similar neighborhood-based use characteristics, yet differ in spatial form, scale, facilities, and activity organization. This allows the study to examine how different combinations of environmental attributes may be associated with restorative-supportive performance in old urban pocket parks.

### 3.2 Research design framework

**Figure 3:**
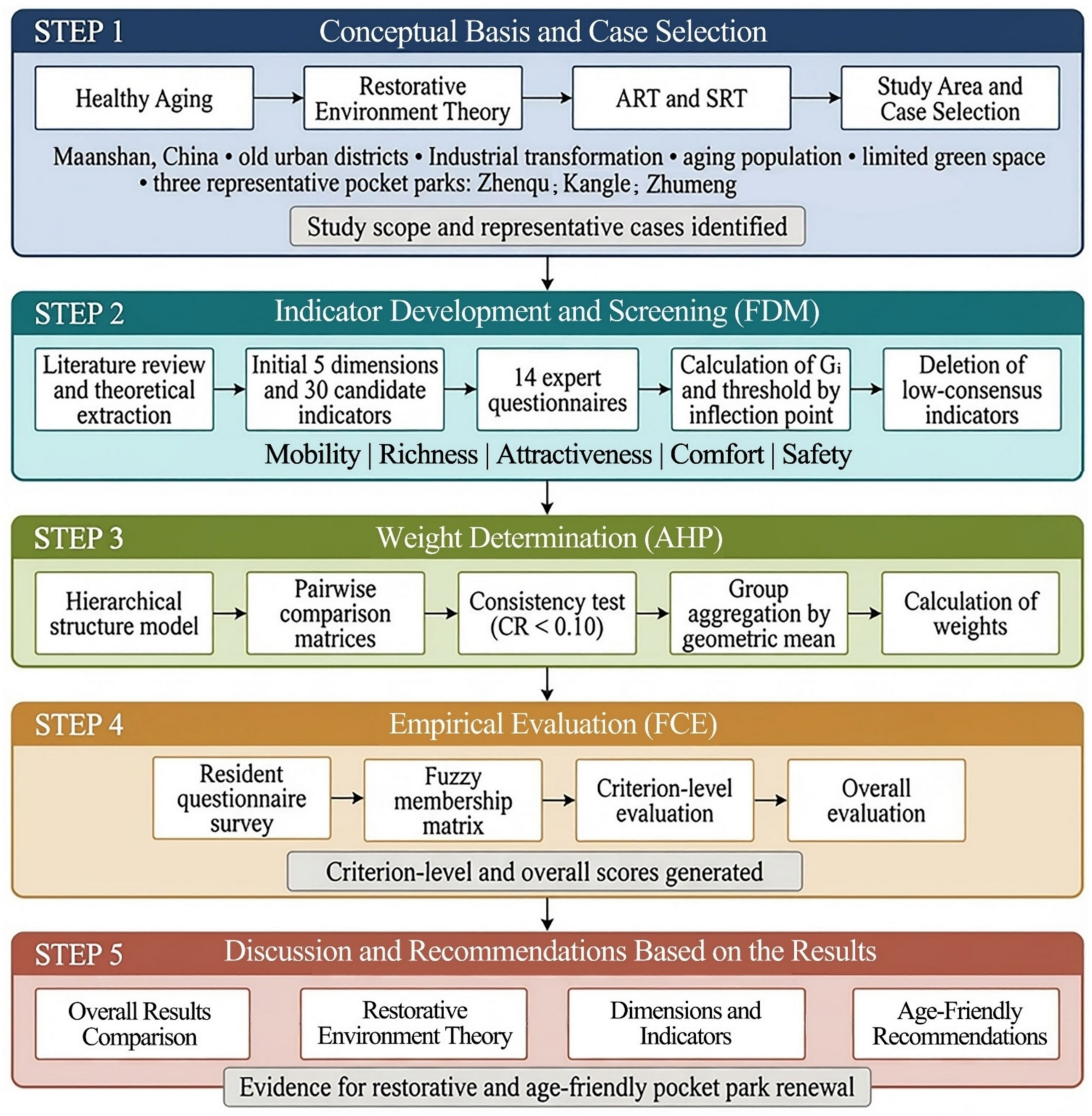
Research framework and workflow.

An integrated research framework was established to assess the restorative potential of pocket parks (Figure 3). The framework consisted of three sequential components: indicator screening, development of the evaluation system, and empirical evaluation. Within this framework, the FDM was used to identify and refine the evaluation indicators, the AHP was applied to derive indicator weights, and the FCE method was employed to comprehensively assess the restorative potential of representative pocket parks.

### 3.3 Preliminary development and operationalization of the evaluation indicator system

ART and SRT provide the main theoretical foundations for restorative environment assessment. ART emphasizes that environmental qualities such as being away, fascination, extent, and compatibility can facilitate the recovery of directed attention (14, 15). SRT suggests that emotional relaxation and stress reduction are more likely to occur in environments perceived as safe, less threatening, and rich in natural elements (16, 17). Based on ART, the PRS has been widely used to measure individuals’ perceived restorativeness of settings, particularly through experiential dimensions such as being away, fascination, extent, and compatibility (19).

However, this study focuses on pocket parks in old urban districts, where older adults are frequent everyday users. In this context, restorative experience is shaped not only by landscape perception, but also by accessibility, safety, age-friendly facilities, staying comfort, and support for daily activities (42, 44, 49). Therefore, this study did not use the PRS as the sole measurement instrument. Instead, it developed a restorative-supportive environmental evaluation framework by integrating ART, SRT, PRS-related research, and studies on age-friendly spaces, pocket parks, and community green-space evaluation (13, 45, 50).

The preliminary indicator system was developed through four steps: theoretical grounding, literature extraction, contextual adaptation, and operationalization. First, ART, SRT, and PRS-related studies were used to identify environmental characteristics associated with attention restoration, stress reduction, environmental attraction, low perceived threat, and compatibility. Second, studies on age-friendly cities, age-friendly public spaces, pocket parks, and community green-space evaluation were reviewed to extract indicators related to older adults’ everyday use, including accessibility, safety, comfort, facility provision, spatial diversity, and landscape perception. Third, based on field investigations in Maanshan’s old urban districts, indicators that were too macro-scale, difficult for users to perceive, or unsuitable for small-scale pocket park evaluation were removed, merged, or transformed. Following the principles of perceptibility, evaluability, comparability, and suitability for questionnaire-based assessment, an initial system comprising five dimensions and 30 candidate indicators was established (Table 2).

**Table 2:** Theoretical basis and preliminary indicators for assessing restorative-supportive environmental quality in pocket parks.

Dimension	Environmental meaning and theoretical link	Indicators	Source of indicators
Mobility (A)	Supports older adults’ access, entry, and continuous movement; related to compatibility in ART.	Pedestrian path walkability (A1)	(49, 50, 77)
		10-min walking accessibility (A2)	(49–51)
		Pavement evenness and ease of walking (A3)	(49, 77)
		Continuity of internal walking routes (A4)	(49, 51, 77)
		Number of entrances and exits (A5)	(49, 51, 77)
		Availability of activity spaces (A6)	(43, 44, 52, 77)
Richness (B)	Refers to diversity in spatial types, facilities, natural elements, and sensory cues; related to extent and soft fascination in ART.	Vegetation structural diversity (B1)	(53–55, 78)
		Seasonal and landscape variation (B2)	(53, 78)
		Diversity of spatial types (B3)	(42, 44, 52, 53)
		Diversity of facility types (B4)	(43, 44, 52, 77)
		Water features (B5)	(16, 17, 76)
		Soundscape quality (B6)	(56, 57)
Attractiveness (C)	Shapes scenic pleasantness, participation willingness, and revisit intention; related to fascination in ART.	Scenic pleasantness (C1)	(15, 42, 53, 79)
		Blue–green landscape characteristics (C2)	(16, 17, 76)
		Environmental cleanliness and maintenance (C3)	(49, 52, 61, 77)
		Accessibility for entry and use (C4)	(49–51)
		Opportunities for activity participation (C5)	(43, 44, 52)
		Revisit intention (C6)	(80)
Comfort (D)	Supports physical relaxation, staying, and low-burden use; related to stress reduction in SRT.	Shading and rain shelter capacity (D1)	(49, 58, 59)
		Seating comfort and age-friendliness (D2)	(49, 60)
		Thermal comfort (D3)	(58, 59)
		Acoustic comfort (D4)	(56, 57)
		Appropriateness of spatial scale (D5)	(15, 42, 53)
		Toilet accessibility and maintenance (D6)	(49, 77)
Safety (E)	Reduces perceived and objective risks; related to low-threat conditions in SRT.	Adequate lighting (E1)	(49, 61, 62)
		Visibility (E2)	(49, 61, 63)
		Safety of internal pathways (E3)	(49, 61, 62)
		External traffic safety (E4)	(49, 51, 62)
		Facility safety (E5)	(49, 60, 77)
		Order maintenance (E6)	(52, 61, 63)

During operationalization, abstract theoretical concepts were translated into concrete environmental elements that older adults could understand and evaluate. For example, compatibility in ART was represented by walkability, pavement evenness, and route continuity; the low-threat condition emphasized in SRT was represented by lighting, visibility, pathway safety, traffic safety, and facility safety; and fascination or soft fascination was reflected through scenic pleasantness, blue–green characteristics, activity participation, and revisit intention. These indicators therefore transformed restorative environment concepts into measurable and comparable evaluation items.

The evaluation system follows a hierarchical multi-criteria structure. The target layer is the assessment of restorative-supportive environmental quality in pocket parks. The criterion layer includes mobility, richness, attractiveness, comfort, and safety, while the indicator layer contains the specific items under each dimension. These dimensions do not form a strict causal chain; rather, they jointly describe the environmental conditions that support older adults’ access, movement, staying, activity, perception, and restorative experience. Based on this structure, FDM was used to screen indicators, AHP to determine dimension and indicator weights, and FCE to conduct the comprehensive evaluation.

Mobility refers to whether older adults can reach pocket parks conveniently and safely, and whether they can move continuously and smoothly within the park and engage in everyday activities once there. This dimension includes not only walkable accessibility at the neighborhood scale, but also the evenness, continuity, and ease of use of internal park pathways. For older adults, surrounding street-network connectivity, walking-path conditions, entrance connections, and internal circulation quality can all influence their willingness to visit, frequency of use, and activity range (49–51). Accordingly, mobility concerns not only access to the park, but also the quality of movement within it.

Richness mainly refers to the diversity of environmental content and spatial experience in pocket parks, including the richness of spatial types, facility types, natural elements, and sensory cues (42, 44, 52, 53). It does not simply refer to species richness in the ecological sense. In restorative experience research, perceived biodiversity has often been found to predict well-being and pleasure more strongly than objective species counts (54, 55). In small urban parks, differences in the composition of lawns, trees, shrubs, and flowers can substantially shape judgments of restorative potential (53).

Attractiveness refers to the extent to which an environment can effortlessly capture attention and sustain interest, thereby fostering attentional recovery and psychological relaxation (15). In pocket parks, more complete seating and resting facilities are generally associated with better restorative outcomes, suggesting that concrete features that support lingering are particularly important (20). Natural soundscapes also matter: birdsong, for example, can enhance perceived restoration, and improved sensory experience may further strengthen overall environmental attractiveness (56, 57).

Comfort first concerns whether the environment is physically tolerable and pleasant, especially in terms of thermal conditions. Shade provision, for instance, can influence older adults’ thermal adaptation and willingness to remain outdoors during hot weather (58, 59). Studies of age-friendly open spaces across different climatic regions in China have confirmed that thermal environmental quality affects older adults’ activity willingness and duration of stay (59). Comfort also depends on the availability of conditions that support staying in place. The accessibility, dimensional suitability, and usability of seating can directly shape whether older adults are willing to sit down and rest (60).

Safety encompasses both objective risk control and older adults’ subjective perceptions of security. In this study, safety refers primarily to whether pocket parks and their immediate surrounding environments can provide low-risk, legible, and comfortable conditions that support older adults’ safe use and staying. Existing studies have shown that older adults are particularly sensitive to visibility, maintenance, and environmental order, all of which can influence their spatial experience and safety judgments (52, 61–63). In pocket parks, visual environmental features may also significantly affect older adults’ perceived safety (64). Accordingly, the safety dimension concerns not only the physical environment within the park, but also the perceived safety support jointly shaped by the park and its adjacent neighborhood environment.

### 3.4 Screening of evaluation indicators

The first step of this study was to establish a candidate indicator system for assessing the restorative quality of pocket parks. The preliminary indicators were derived from three sources: a review of the relevant literature, field investigation, and the observed characteristics of pocket parks in old urban districts. However, reliance on the research team’s judgment alone would have risked introducing subjective bias. In addition, experts from different disciplinary backgrounds may interpret the functions and qualities of pocket parks in different ways. It was therefore necessary to screen the candidate indicators through expert consensus before proceeding to weight derivation.

To obtain a more robust and stable consensus, this study employed the FDM. By incorporating fuzzy numbers into the conventional Delphi procedure, FDM represents expert judgment as an interval together with its most plausible value, thereby capturing expert cognition more faithfully (65). Traditional Delphi methods typically require multiple rounds of consultation and feedback, making the process time-consuming. Moreover, experts are often asked to provide a single-point score, which constrains their ability to express uncertainty in their judgments. In the evaluation of public-space environments, however, judgments regarding indicator importance are inherently fuzzy and uncertain. If only single-value ratings are used, the flexibility and divergence embedded in expert opinion may be underestimated.

Based on these considerations, 14 experts were selected for this study. The selection criteria required the experts to have relevant backgrounds in design, landscape planning, urban regeneration, healthy human settlements, or public-space evaluation; practical or research experience in older adult community planning, age-friendly design, age-friendly public spaces, or health-oriented environmental evaluation; and direct involvement within the past 3 years in research, planning and design, community service, or public-space assessment related to older adults.

In the FDM questionnaire, each indicator was rated on a 0–10 scale according to its importance, with higher scores indicating greater importance. For each indicator, experts were asked to provide three judgment values: the minimum acceptable value, the most appropriate value, and the maximum acceptable value. They could also propose modifications, merging, or supplementary indicators where necessary. The expert responses were subsequently aggregated and processed using fuzzy methods to derive the consensus evaluation value for each indicator.

To further determine whether effective expert consensus had been reached, this study constructed the conservative triangular fuzzy number $C_{i}=(C_{L}^{i},C_{M}^{i},C_{U}^{i})$ and the optimistic triangular fuzzy number $O_{i}=(O_{L}^{i},O_{M}^{i},O_{U}^{i})$ for each indicator.

When there was no obvious overlap between the two triangular fuzzy numbers, the expert judgments were considered relatively consistent. When an acceptable overlap existed, the consensus value *G_i_* was calculated using the intersection point of the membership functions.

Specifically, when $C_{U}^{i}>O_{L}^{i}$ , the consensus value was calculated as follows:


$$G_{i}=\frac{C_{U}^{i}(O_{M}^{i}-O_{L}^{i})+O_{L}^{i}(C_{U}^{i}-C_{M}^{i})}{\left(O_{M}^{i}-O_{L}^{i})+(C_{U}^{i}-C_{M}^{i}\right)}$$

The value of *G_i_* reflects the overall degree of expert consensus on the importance of the *i*-th indicator. The indicators were then ranked according to their *G_i_* values, and the screening threshold was determined using the inflection-point method.

This study completed the indicator screening through one round of Fuzzy Delphi consultation. Unlike the traditional Delphi method, which usually requires multiple rounds of anonymous feedback, the Fuzzy Delphi Method represents the uncertainty range of expert judgments using fuzzy numbers and integrates expert opinions through group fuzzy numbers, membership-function intersections, and consensus values (36). Under the predefined 0–10 importance rating scale, fuzzy judgment procedure, and threshold rule based on *G_i_* ranking and the inflection-point method, one round of expert consultation was sufficient for the purposes of this study. Indicators below the threshold were considered to have insufficient consensus and retention necessity, whereas indicators reaching or exceeding the threshold were retained for subsequent AHP weight calculation.

### 3.5 Determination of indicator weights

The AHP, proposed by Thomas L. Saaty, is a multi-criteria decision-making method that integrates qualitative judgment with quantitative analysis (66–69). By constructing a hierarchical decision structure and conducting pairwise comparisons among elements at the same level, AHP derives the relative weights of individual factors and evaluates the reliability of these judgments through a consistency test. In this study, AHP was used to assign weights to the evaluation indicators retained after FDM screening, thereby identifying the relative importance of each dimension and indicator in the assessment of restorative-supportive environments in pocket parks. The weighting process was based on pairwise comparison judgments provided by the same 14 experts who participated in the FDM procedure. Thus, the AHP results primarily reflect the relative importance of the dimensions and indicators from a professional knowledge perspective, rather than directly representing the preferences of older residents. Older residents’ subjective evaluations were incorporated in the subsequent FCE stage, where their questionnaire responses were used to construct the membership matrix for each indicator.

### 3.6 Resident questionnaire survey

The Fuzzy Comprehensive Evaluation (FCE) method is suitable for evaluation problems involving multiple indicators, strong subjectivity, and fuzzy boundaries between evaluation grades. Given the subjective nature of older adults’ perceptions of the restorative-supportive environmental quality of pocket parks, this study employed FCE to conduct a comprehensive evaluation of the three pocket parks. Specifically, the evaluation questionnaire was developed based on the indicators retained after FDM screening, and the comprehensive measurement was conducted by incorporating the indicator weights derived from AHP. A five-level evaluation scale was adopted, comprising “very good,” “good,” “average,” “poor,” and “very poor,” to capture older respondents’ subjective judgments of each environmental indicator.

In this study, “older respondents” referred to park users aged 60 years and above who were engaging in activities or temporarily staying in the three pocket parks during the survey period, and who were able to complete the questionnaire independently or with explanation from the research team. The questionnaire recorded only basic information, including gender, age group, time of park visit, visit frequency, duration of each stay, and main activity type. To reduce the response burden on older respondents and protect personal privacy, this study did not collect sensitive information such as income, specific residential address, identity documents, or medical history. Nor did it conduct medical or scale-based measurements of mobility, health status, or functional ability.

During fuzzy data processing, the proportion of respondents assigned to each of the five evaluation grades was first calculated for each indicator and then converted into membership degrees. For the *i*-th indicator, if the number of respondents selecting the *j*-th evaluation grade is *n_ij_*, and the total number of valid questionnaires for the park is *N*, then the membership degree of this indicator to the *j*-th evaluation grade is the FCE calculation procedure is shown in Equations 1-4:


$$r_{\mathrm{ij}}=\frac{n_{\mathrm{ij}}}{N}$$ (1)

A single-factor fuzzy evaluation matrix *R* was thereby formed. Subsequently, the indicator weight vector *W* obtained from AHP was combined with the fuzzy evaluation matrix *R* to obtain the comprehensive membership vector:


$$B=W\times R=(b_{1},b_{2},b_{3},b_{4},b_{5})$$ (2)

where *b_j_* denotes the comprehensive membership degree of the evaluation object belonging to the *j*-th evaluation grade. To further obtain comparable comprehensive scores, this study set the grade score vector as:


S=(95,85,75,65,57.5) (3)

corresponding, respectively, to the five evaluation grades of “very good,” “good,” “average,” “poor,” and “very poor.” The final comprehensive score was calculated as:


$$F=B\times S^{T}$$ (4)

The final comprehensive scores *F* were used to compare the relative restorative-supportive performance of the three pocket parks. The dimension-level and indicator-level results were further analyzed to identify the performance characteristics and main weaknesses of each park.

### 3.7 Robustness testing and statistical analysis

To examine the robustness of the AHP–FCE results, a criterion-level weight sensitivity analysis was performed. Because alternative rankings in multi-criteria decision-making may vary with changes in criterion weights (70), a one-at-a-time perturbation approach was adopted. The weight of each criterion-level dimension was increased and decreased by 10 and 20%, respectively, while the remaining criterion weights were proportionally normalized to maintain a total weight of 1. The local indicator weights within each dimension were kept unchanged. The comprehensive scores and ranking order of the three pocket parks were then recalculated under each perturbation scenario.

An indicator-level non-parametric comparison was also conducted to test whether the three parks differed in their score profiles. Because the survey data were retained as park-specific frequency summaries and aggregated indicator-level scores rather than individual respondent-level records, the analysis was conducted at the indicator-score profile level. The 25 retained indicators were treated as repeated analytical units, and the Friedman test was used to compare the three parks across the evaluation system (71). When significant differences were detected, pairwise Wilcoxon signed-rank tests were performed, with Holm correction applied for multiple comparisons (72, 73). Statistical significance was set at *p* < 0.05. Accordingly, this analysis was used to support the comparison of park-level performance profiles across the three cases, while the interpretation remained focused on aggregated indicator-level results.

## 4 Results

### 4.1 Results of indicator screening

**Figure 4:**
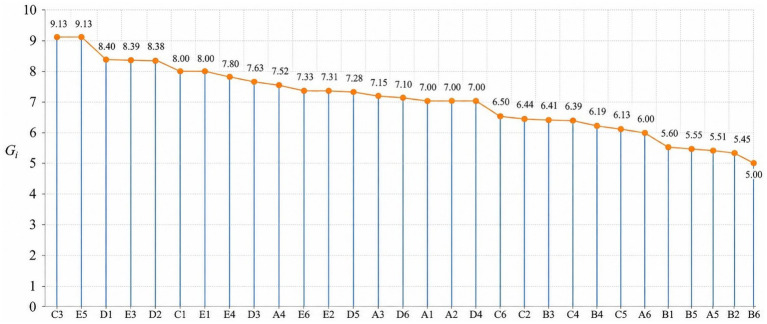
Line chart of *G_i_* values for each evaluation indicator.

The FDM questionnaire was distributed to 14 experts on 8 October 2025, and all responses were collected by 20 October 2025. After calculating the consensus score for each candidate indicator, the indicators were ranked in descending order of their Gi values and plotted as a line graph (Figure 4). The cutoff threshold for indicator retention was set at 6.00. Indicators with *G_i_* values below 6.00 were considered to have relatively low expert consensus and were excluded from the evaluation system.

Accordingly, five indicators were removed: Number of entrances and exits (A5), Vegetation structural diversity (B1), Seasonal and landscape variation (B2), Water features (B5), and Soundscape quality (B6).

The FDM results retained 25 indicators and excluded 5 indicators (Table 3). Within the Mobility dimension, the retained indicators were Pedestrian path walkability (A1), 10-min walking accessibility (A2), Pavement evenness and ease of walking (A3), Continuity of internal walking routes (A4), and Availability of activity spaces (A6). Within Richness, the retained indicators were Diversity of spatial types (B3) and Diversity of facility types (B4). Within Attractiveness, the retained indicators were Scenic pleasantness (C1), Blue–green landscape characteristics (C2), Environmental cleanliness and maintenance (C3), Accessibility for entry and use (C4), Opportunities for activity participation (C5), and Revisit intention (C6). Within Comfort, the retained indicators were Shading and rain shelter capacity (D1), Seating comfort and age-friendliness (D2), Thermal comfort (D3), Acoustic comfort (D4), Appropriateness of spatial scale (D5), and Toilet accessibility and maintenance (D6). Within Safety, the retained indicators were Adequate lighting (E1), Visibility (E2), Safety of internal pathways (E3), External traffic safety (E4), Facility safety (E5), and Order maintenance (E6).

**Table 3:** FDM questionnaire results.

Dimension	Indicators	Geometric mean	*C_U_*	*C_L_*	*O_U_*	*O_L_*	*G_i_*	Result
Mobility (A)	A1	4.81	7.00	2.00	10.00	7.00	7.00	Retained
	A2	4.62	7.00	3.00	10.00	7.00	7.00	Retained
	A3	4.75	10.00	1.00	10.00	5.00	7.15	Retained
	A4	4.16	9.00	2.00	10.00	7.00	7.52	Retained
	A5	3.74	6.00	2.00	9.00	5.00	5.51	Delete
	A6	3.45	6.00	2.00	10.00	6.00	6.00	Retained
Richness (B)	B1	3.83	6.00	2.00	10.00	5.00	5.60	Delete
	B2	2.62	6.00	1.00	9.00	5.00	5.45	Delete
	B3	3.57	7.00	2.00	10.00	6.00	6.41	Retained
	B4	3.40	8.00	2.00	10.00	5.00	6.19	Retained
	B5	3.69	6.00	2.00	9.00	5.00	5.55	Delete
	B6	3.79	5.00	3.00	9.00	5.00	5.00	Delete
Attractiveness (C)	C1	4.83	8.00	2.00	10.00	8.00	8.00	Retained
	C2	4.05	7.00	2.00	10.00	6.00	6.44	Retained
	C3	4.66	10.00	1.00	10.00	9.00	9.13	Retained
	C4	4.13	8.00	2.00	10.00	5.00	6.39	Retained
	C5	4.15	7.00	2.00	10.00	5.00	6.13	Retained
	C6	4.29	7.00	3.00	10.00	6.00	6.50	Retained
Comfort (D)	D1	5.20	10.00	3.00	10.00	8.00	8.40	Retained
	D2	4.65	10.00	2.00	10.00	8.00	8.38	Retained
	D3	3.65	10.00	1.00	10.00	7.00	7.63	Retained
	D4	4.45	7.00	1.00	10.00	7.00	7.00	Retained
	D5	3.87	8.00	1.00	10.00	7.00	7.28	Retained
	D6	4.25	10.00	2.00	10.00	5.00	7.10	Retained
Safety (E)	E1	4.96	8.00	3.00	10.00	8.00	8.00	Retained
	E2	4.39	8.00	2.00	10.00	7.00	7.31	Retained
	E3	4.86	10.00	2.00	10.00	8.00	8.39	Retained
	E4	4.43	10.00	2.00	10.00	7.00	7.80	Retained
	E5	4.92	10.00	1.00	10.00	9.00	9.13	Retained
	E6	4.59	8.00	3.00	10.00	7.00	7.33	Retained

### 4.2 Results of indicator weighting

Building on the FDM screening results, an AHP hierarchical structure model was further developed to determine the relative weights of the dimensions and indicators used to assess the restorative environment of pocket parks. The model comprised two levels: a dimension level and an indicator level. The dimension level included five dimensions—Mobility, Richness, Attractiveness, Comfort, and Safety—whereas the indicator level consisted of the 25 retained indicators (Table 4). Based on this structure, an expert pairwise-comparison questionnaire was developed, and the Saaty 1–9 scale was employed to judge relative importance.

**Table 4:** Weights of evaluation indicators.

Dimension	*W_a_*	Indicator	*W_cnb_*	*W_c_*
Mobility (A)	0.1568	A1	0.1652	0.0259
		A2	0.1690	0.0265
		A3	0.3404	0.0534
		A4	0.2230	0.0350
		A6	0.1025	0.0161
Richness (B)	0.0605	B3	0.5965	0.0361
		B4	0.4035	0.0244
Attractiveness (C)	0.0591	C1	0.1487	0.0088
		C2	0.0581	0.0034
		C3	0.3533	0.0209
		C4	0.2355	0.0139
		C5	0.0995	0.0059
		C6	0.1049	0.0062
Comfort (D)	0.1697	D1	0.1931	0.0328
		D2	0.1872	0.0318
		D3	0.0952	0.0161
		D4	0.0851	0.0144
		D5	0.2217	0.0376
		D6	0.2179	0.0370
Safety (E)	0.5539	E1	0.0553	0.0306
		E2	0.0639	0.0354
		E3	0.2150	0.1191
		E4	0.2180	0.1208
		E5	0.3195	0.1770
		E6	0.1282	0.0710

W_a_ denotes the weight of each criterion-level dimension relative to the target level. W_cnb_ denotes the weight of each indicator-level item relative to its corresponding criterion level. W_c_ denotes the global weight of each indicator-level item relative to the target level.

The AHP questionnaire was distributed to the same 14 experts on 27 October 2025, and all questionnaires were returned by 10 November 2025. After collection, individual pairwise comparison matrices were constructed for each expert at both the dimension level (A–E) and the indicator level (A1–A4, A6; B3–B4; C1–C6; D1–D6; and E1–E6). The individual matrices were first inspected for consistency, and the expert judgments were then aggregated using the geometric mean to construct group judgment matrices, a classical aggregation procedure widely used in group AHP and group decision-making research (74, 75). The consistency of the aggregated group matrices was subsequently tested, and all group matrices satisfied the consistency requirement (*CR*<0.10). On this basis, the dimension weights (*W_a_*), local weights (*W_cnb_*), and global weights (*W_c_*) were calculated to provide the weighting input for the subsequent FCE-based comprehensive evaluation.

**Figure 5:**
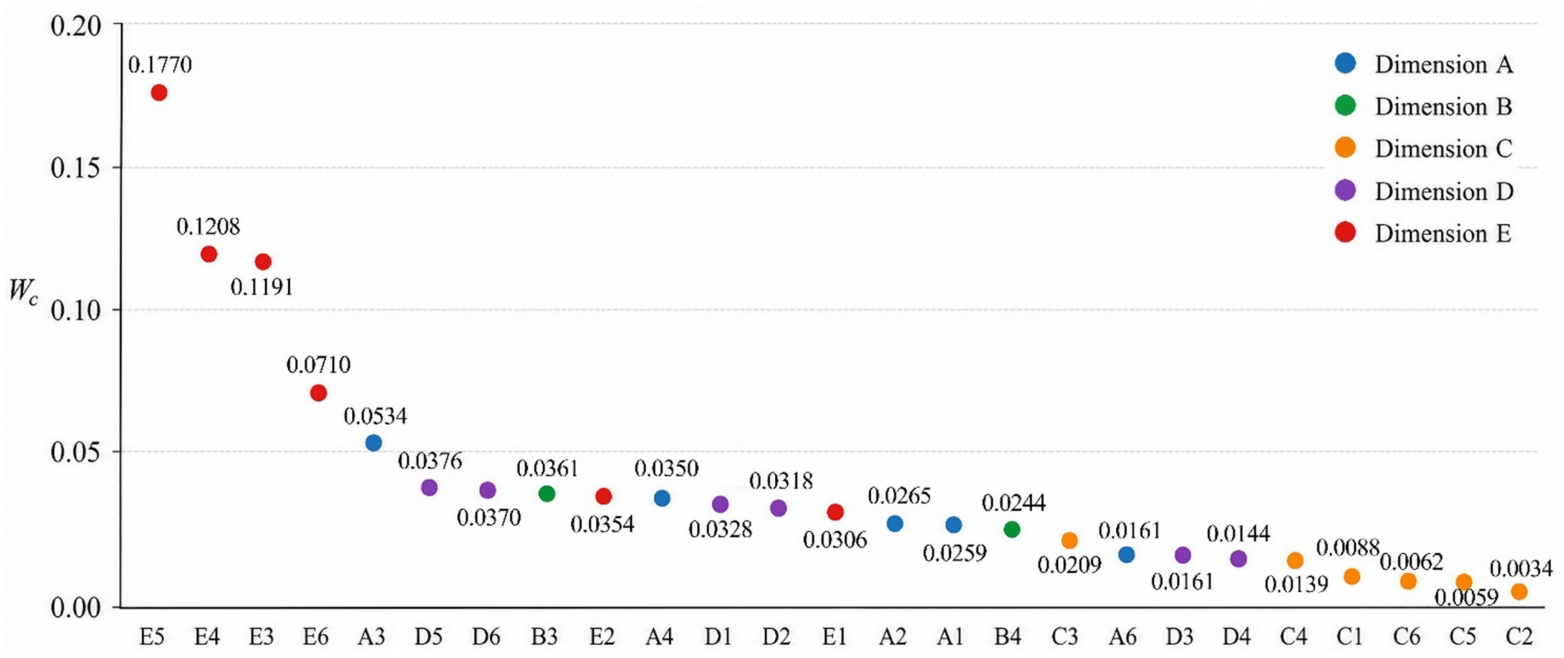
Scatter plot of indicator weights.

Based on the composite weights derived from the Analytic Hierarchy Process, the ranking values of the evaluation indicators for the restorative environment of pocket parks were obtained and visualized in a scatter plot (Figure 5).

### 4.3 FCE questionnaire results

In November 2025, a paper-based questionnaire survey was conducted through on-site distribution and on-site collection. A total of 300 questionnaires were distributed, and 278 valid questionnaires were returned, resulting in an effective response rate of 92.7%. Among them, 100 questionnaires were distributed in Zhenqu Pocket Park, with 94 valid responses collected; 100 were distributed in Kangle Pocket Park, with 91 valid responses collected; and 100 were distributed in Zhumeng Pocket Park, with 93 valid responses collected.

Given that the respondents were older adults, the questionnaire was designed to reduce the burden of on-site completion and to protect personal privacy. It did not collect sensitive information such as income, specific residential address, or identity documents. Instead, it recorded basic information related to sample composition and park-use behavior, including gender, age group, time of park visit, visit frequency, duration of each stay, and main activity type. On this basis, the demographic and park-use characteristics of the older respondents were summarized.

The respondents were mainly concentrated in the 60–69 and 70–79 age groups, while those aged 80 years and above accounted for only a small proportion. Most respondents also reported short travel times to the parks and relatively high visit frequency, indicating that the three pocket parks were closely embedded in older adults’ everyday activity spaces and had strong neighborhood-based use characteristics. Based on the completed questionnaires, the proportion of responses assigned to each evaluation grade was calculated for every indicator. These proportions were then used to construct the single-factor fuzzy evaluation matrix, from which the indicator-level scores of the three pocket parks were obtained (Table 5).

**Table 5:** Indicator-level scores for the three pocket parks.

Indicators	Zhenqu pocket park	Kangle pocket park	Zhumeng pocket park
A1	81.277	79.670	78.306
A2	81.277	79.670	76.640
A3	82.739	81.154	79.785
A4	81.277	79.670	78.306
A6	77.979	76.456	74.973
B3	79.628	79.670	76.640
B4	77.979	76.456	74.973
C1	79.628	78.269	76.640
C2	76.223	74.670	73.602
C3	81.277	81.154	78.306
C4	77.979	76.456	74.973
C5	76.223	74.670	73.602
C6	77.979	76.456	74.973
D1	81.277	79.670	78.306
D2	81.277	79.670	78.306
D3	77.979	76.456	74.973
D4	76.223	74.670	73.602
D5	79.628	79.670	76.640
D6	77.979	76.456	74.973
E1	79.628	79.670	76.640
E2	79.628	79.670	76.640
E3	82.739	81.154	79.785
E4	82.739	81.154	79.785
E5	83.697	82.555	81.129
E6	81.277	81.154	78.306

### 4.4 Results of robustness testing and statistical comparison

Using the indicator-level scores and AHP-derived weights, the baseline AHP–FCE results showed that Zhenqu Pocket Park obtained the highest comprehensive score (81.39), followed by Kangle Pocket Park (80.26) and Zhumeng Pocket Park (78.46), indicating a clear performance gradient among the three parks.

The weight sensitivity analysis showed that the comprehensive evaluation results remained stable under all weight-perturbation scenarios (Table 6). Under the ±10 and ±20% criterion-level perturbation scenarios, the comprehensive scores ranged from 81.12 to 81.67 for Zhenqu Pocket Park, from 79.97 to 80.55 for Kangle Pocket Park, and from 78.16 to 78.75 for Zhumeng Pocket Park. The ranking order remained unchanged across all perturbation scenarios. The equal-weight robustness checks also produced the same ranking pattern, suggesting that the comparative results were not driven by a single dominant criterion weight.

**Table 6:** Results of weight sensitivity analysis and equal-weight robustness checks.

Scenario	Zhenqu	Kangle	Zhumeng	Ranking
Baseline AHP weights	81.39	80.26	78.46	Zhenqu > Kangle > Zhumeng
±10 and ±20% criterion-level weight perturbations	81.12–81.67	79.97–80.55	78.16–78.75	Unchanged
Equal criterion weights	80.29	79.20	77.30	Unchanged
Equal indicator weights	79.82	78.65	76.83	Unchanged

The indicator-level non-parametric comparison further confirmed significant differences in the score profiles of the three parks across the 25 retained indicators (Table 7). The Friedman test indicated a significant difference among the three parks, χ^2^(2) = 43.28, *p* < 0.001, with Kendall’s W = 0.866. Pairwise Wilcoxon signed-rank tests with Holm correction showed significant differences for all pairwise comparisons, with all adjusted *p* values below 0.001. These findings provide additional statistical support for the ranking pattern of Zhenqu Pocket Park > Kangle Pocket Park > Zhumeng Pocket Park at the indicator-score profile level.

**Table 7:** Indicator-level non-parametric comparison among the three pocket parks.

Test/comparison	Statistic	Adjusted *p*-value	Interpretation
Friedman test	χ^2^(2) = 43.28	*p* < 0.001	Significant difference among the three parks
Zhenqu vs. Kangle	Wilcoxon statistic = 10.00	*p* < 0.001	Significant
Zhenqu vs. Zhumeng	Wilcoxon statistic = 0.00	*p* < 0.001	Significant
Kangle vs. Zhumeng	Wilcoxon statistic = 0.00	*p* < 0.001	Significant

## 5 Discussion

### 5.1 Identifying and defining the core dimensions of restorative environments

From the perspective of restorative environment theory, the five dimensions identified in this study should not be understood as a direct application of existing restorative scales. Rather, they represent a contextual translation of ART, SRT, age-friendly spatial needs, and the everyday use context of pocket parks in old urban districts. ART emphasizes the role of being away, fascination, extent, and compatibility in attentional recovery, while SRT highlights the contribution of low-threat natural environments to emotional relaxation and stress reduction (14–17). Restorative scales such as the PRS further suggest that abstract theoretical mechanisms need to be translated into perceptible and evaluable environmental characteristics (19). However, in small-scale, neighborhood-based, and frequently used pocket parks, restorative potential is not determined solely by natural elements or the amount of greenery. It also depends on whether older adults can reach the site smoothly, use it safely, stay comfortably, and experience moderate environmental variation and attraction. Accordingly, mobility, richness, attractiveness, comfort, and safety can be understood as operational dimensions through which classical restorative theories are adapted to older adults’ everyday use of pocket parks.

Building on this interpretation, the conceptual contribution of this study lies in extending restorative environment theory from a framework centered primarily on nature exposure and perceived restorativeness to an evaluation framework for restorative-supportive environments. Unlike the PRS, which mainly measures individuals’ subjective perception of environmental restorativeness, the framework proposed in this study does not directly assess psychological or physiological restoration outcomes that have already occurred. Instead, it evaluates whether pocket parks possess the environmental conditions that can support restorative experiences among older adults. In this sense, the restorative environmental quality examined here is more accurately understood as restorative-supportive environmental quality. It refers to the comprehensive environmental potential of pocket parks to support attention restoration, stress reduction, emotional relaxation, everyday activities, and social interaction through safe accessibility, comfortable conditions for staying, environmental richness, and experiential attraction.

Within this framework, the five dimensions play different but complementary roles. Mobility, safety, and comfort constitute the basic conditions for restorative experience. Mobility determines whether older adults can conveniently reach and smoothly use the park; safety affects whether they are willing to enter, stay, and develop environmental trust; and comfort concerns bodily perceptual conditions such as shading, seating, thermal environment, acoustic environment, and spatial scale (59). By contrast, richness and attractiveness function more as enhancement conditions. Richness provides more activity choices and sensory stimuli through diverse spaces, facilities, and environmental content, whereas attractiveness influences continued use and revisit intention through scenic pleasantness, blue–green landscape characteristics, environmental maintenance, and opportunities for activity participation (49, 50, 54). Taken together, the restorativeness of pocket parks in old urban districts should not be equated simply with the presence of greenery. Rather, it should be understood as the joint outcome of everyday accessibility, risk control, physical comfort, environmental variation, and experiential attraction.

### 5.2 Determining restorative indicators of greater importance for older adults

The AHP weights in this study were derived from pairwise comparison judgments made by 14 experts. Therefore, the weighting results should be interpreted primarily as expert-based priorities regarding the components of restorative-supportive environmental quality in pocket parks in old urban districts, rather than as a direct or complete representation of older residents’ own preferences. Older residents’ subjective evaluations were incorporated into the assessment process through the FCE questionnaire, which was used to construct the membership matrix for each indicator and was then combined with expert-derived weights to generate the comprehensive evaluation results. Accordingly, the weights reported in this study are better understood as an expert-knowledge-guided evaluation priority structure, rather than as a preference ranking generated directly by older residents.

Within this weighting structure, safety received the highest weight, followed by comfort and mobility, whereas richness and attractiveness received relatively lower weights. This pattern may reflect the combined influence of the spatial conditions of old urban districts and the use characteristics of older adults. Pocket parks in old urban districts are usually embedded in high-density built environments, where road connections, aging facilities, insufficient lighting, and uneven maintenance may directly affect older adults’ experience of use. At the same time, older adults are often more sensitive to fall risk, route continuity, facility safety, and comfort for staying. Previous studies have also shown that micro-scale environmental conditions, such as safety, accessibility, and comfort, can influence older adults’ willingness to engage in outdoor activities, frequency of space use, and environmental perception (45, 60, 76). The relatively high weights assigned to safety, mobility, and comfort therefore suggest that these dimensions function as basic conditions for restorative experiences among older adults.

By contrast, the relatively lower weights of richness and attractiveness should not be interpreted as indicating that these dimensions are unimportant. Rather, in the expert judgments of this study, they appear to function more as experience-enhancing conditions than as basic thresholds for entry and stay. In other words, after safety, accessibility, and comfort are adequately secured, environmental richness and attractiveness may further promote staying, participation, revisitation, and restorative experience. This interpretation is broadly consistent with previous pocket park studies showing that spatial composition, activity conditions, and opportunities for staying can influence health-promoting use (44, 45).

Taken together, the weighting results suggest that age-friendly restorative environment development in pocket parks should first secure baseline conditions related to safety, movement, and comfort, and then enhance restorative value through environmental enrichment and experiential attraction. Safety, mobility, and comfort determine whether pocket parks can serve as usable and reliable everyday spaces for older adults, whereas richness and attractiveness influence whether these spaces can move beyond basic usability to become everyday environments with sustained appeal and restorative benefits.

### 5.3 Comparing restorative-supportive performance across the three pocket parks

The results provide more robust evidence for comparing the restorative-supportive performance of the three pocket parks. Because the three cases were selected from the same old urban districts and belong to the same municipal pocket park development system, they share similar policy contexts, neighborhood service functions, and everyday use characteristics. This shared context allows the comparison to reveal how differences in spatial organization, facility provision, and environmental experience may shape restorative-supportive performance.

The comprehensive scores showed a clear performance gradient, with Zhenqu Pocket Park performing best, followed by Kangle Pocket Park and Zhumeng Pocket Park. This pattern was supported by the weight sensitivity analysis, which showed that the ranking remained unchanged under all criterion-level perturbation scenarios. The indicator-level non-parametric comparison further confirmed significant differences in the score profiles of the three parks across the 25 retained indicators. These findings suggest that the observed differences were not merely minor fluctuations in FCE scores, but reflected relatively stable differences in park-level restorative-supportive performance.

The three parks also shared a common structural pattern. Safety-related indicators generally performed well, whereas richness- and attractiveness-related indicators remained comparatively weak. This suggests that pocket park development in old urban districts has responded relatively well to basic safety and everyday usability needs, but still requires improvement in spatial layering, environmental variation, activity organization, and sustained experiential attraction. Specifically, Zhenqu Pocket Park showed stronger performance in safety, movement, comfort, and facility-related conditions; Kangle Pocket Park showed a more balanced but less distinctive profile; and Zhumeng Pocket Park performed relatively weakly in several richness, attractiveness, and comfort-related indicators. These results indicate that restorative improvement in old urban pocket parks requires integrated optimization of accessibility, safety, comfort, environmental diversity, and experiential appeal.

### 5.4 Age-friendly renewal strategies for pocket parks in old urban districts

Based on the FCE results, weight sensitivity analysis, and indicator-level statistical comparison, the age-friendly renewal of pocket parks in old urban districts should focus on three aspects: securing basic support, enhancing restorative experience, and implementing differentiated optimization strategies.

First, basic conditions related to safety, mobility, and comfort should be prioritized. This includes maintaining continuous and even park pathways, improving the connection between entrances and surrounding roads, enhancing night-time lighting and visual accessibility, and regularly maintaining seating, fitness equipment, railings, paving, and drainage facilities. Additional shading, rain shelters, age-friendly seating, and quiet resting areas should also be provided. For older adults, these measures affect not only whether they can enter and move through the park smoothly, but also whether they are willing to stay and develop stable everyday use habits.

Second, deficiencies in environmental richness and attractiveness should be addressed to enhance restorative experience. Renewal should not be limited to increasing greenery or adding facilities; rather, it should focus on improving spatial layering, activity choices, and sensory experience. Measures such as multilayered planting, seasonal landscapes, perceptible blue–green elements, low-intensity activity facilities, neighborhood interaction nodes, and quiet resting spaces can help improve older adults’ willingness to stay, revisit intention, and restorative experience. At the same time, excessive activity-oriented development should be avoided. Quiet, low-disturbance spaces that allow both solitude and social interaction should be preserved.

Third, renewal strategies should be differentiated according to the statistically supported performance gradient and the main weaknesses of each park. Zhenqu Pocket Park could further enhance landscape interest, activity participation, and experiential attractiveness, building on its relatively strong performance in safety, movement, comfort, and facility-related conditions. Kangle Pocket Park showed a balanced but less distinctive profile, suggesting the need to improve planting layers, staying comfort, and the perceptibility of natural elements. Zhumeng Pocket Park should prioritize entrance organization, walking continuity, facility diversity, environmental maintenance, and comfort-related support. Overall, pocket park renewal in old urban districts should move beyond the provision of green and usable spaces toward quality-oriented improvements that integrate safe accessibility, comfortable staying conditions, environmental richness, and sustained experiential appeal.

### 5.5 Study limitations

This study has several limitations. First, the AHP weights were derived from pairwise comparison judgments made by 14 experts. Although the experts had relevant experience in age-friendly design, health-oriented environmental evaluation, and aging-related research or practice, the weights mainly reflect a professional knowledge perspective and cannot be fully equated with older residents’ own preferences. Second, the survey recorded only basic demographic and park-use information, including gender, age group, visit time, visit frequency, duration of stay, and main activity type, but did not systematically collect data on mobility capacity, chronic disease status, self-rated health, or functional ability. Therefore, differences among older adults with different health or functional profiles could not be further examined. Third, the empirical analysis included only three pocket parks in the old urban districts of Maanshan, all within the same municipal pocket park development system. These cases reflect typical features of old urban pocket parks, including everyday accessibility, neighborhood-based use, and age-friendly renewal. However, their shared development context and limited number mean that the findings should be interpreted with caution. The comparison is therefore most useful for understanding how different spatial configurations may relate to restorative-supportive performance within this case context, while broader conclusions require further validation using larger and more diverse samples. Although weight sensitivity analysis and indicator-level non-parametric comparison were added to strengthen the robustness of the findings, the comparative data were retained as park-specific frequency summaries and aggregated indicator-level scores rather than individual respondent-level records. The statistical results should therefore be interpreted as evidence of differences in park-level performance profiles. Future studies should retain individual questionnaire records to enable respondent-level statistical testing, subgroup analysis, and more refined modelling based on older adults’ demographic, health, and functional characteristics.

## 6 Conclusion

This study examined three representative pocket parks located in the old urban districts of Maanshan and, within the dual framework of healthy aging and restorative environment theory, developed and applied an integrated evaluation approach combining the FDM, the AHP, and FCE to systematically assess restorative environmental quality. The findings demonstrate that the resulting evaluation system, consisting of five dimensions and 25 indicators, effectively captures the key characteristics of restorative environments in pocket parks situated in old urban districts. Among the five dimensions, safety carried the greatest weight, followed by comfort and mobility, whereas richness and attractiveness received lower weights. Although all three case parks exhibited a certain degree of restorative-supportive potential, their comprehensive scores showed a clear and statistically supported performance gradient at the indicator-score profile level, with Zhenqu Pocket Park performing best, followed by Kangle Pocket Park and Zhumeng Pocket Park. Taken together, these findings suggest that enhancing the restorative quality of pocket parks in old urban districts cannot rely solely on adding greenery or increasing the number of facilities. Rather, it requires the coordinated optimization of safety provision, walkability support, comfort enhancement, richness, and experiential attractiveness. By foregrounding the everyday use contexts of older adults, this study offers an operational analytical framework for evaluating restorative environments in pocket parks located in old urban districts, while also providing empirical evidence to inform the development of age-friendly communities and small-scale spatial regeneration.

## Data Availability

The original contributions presented in the study are included in the article/Supplementary material, further inquiries can be directed to the corresponding author.

## References

[1] United Nations, Department of Economic and Social Affairs, Population Division World population prospects 2022: summary of results (2022). Available online at: https://www.un.org/development/desa/pd/sites/www.un.org.development.desa.pd/files/wpp2022_summary_of_results.pdf (Accessed December 16, 2025).

[2] Beard JR Officer A de Carvalho IA Sadana R Pot AM Michel J-P . The world report on ageing and health: a policy framework for healthy ageing. Lancet. (2016) 387:2145–54. doi: 10.1016/S0140-6736(15)00516-4, 26520231 PMC4848186

[3] United Nations General Assembly United Nations Decade of Healthy Ageing (2021–2030) (2020). Available online at: https://docs.un.org/en/A/RES/75/131 (Accessed December 18, 2025).

[4] World Health Organization Decade of healthy ageing: plan of action (2020). Available online at: https://www.who.int/publications/m/item/decade-of-healthy-ageing-plan-of-action (Accessed December 18, 2025).

[5] Hu LL Glavin YFW. Integrating health and care for older people in China: what has been accomplished? What is next? Int J Integr Care. (2023) 23:16. doi: 10.5334/ijic.7598, 36967837 PMC10038113

[6] Zhang Y van Dijk T Weitkamp G van den Berg AE. The relationship between park design and seniors’ use of green spaces in Xi’an, China. J Public Space. (2023) 8:21–40. doi: 10.32891/jps.v8i2.1348

[7] Cui H Maliki N Wang Y. The role of urban parks in promoting social interaction of older adults in China. Sustainability. (2024) 16:2088. doi: 10.3390/su16052088

[8] Wang X Rodiek S. Older adults' preference for landscape features along urban park walkways in Nanjing, China. Int J Environ Res Public Health. (2019) 16:3808. doi: 10.3390/ijerph16203808, 31658651 PMC6843449

[9] Yang B Hong B. Pocket park in urban regeneration of China: policy and perspective. City Environ Interact. (2023) 19:100109. doi: 10.1016/j.cacint.2023.100109

[10] Xinhua China unveils plan for 15-min “living circles” in cities (2023). Available online at: https://english.news.cn/20230712/6f71c5fc8b3643a9a2cace89d9a663f9/c.html (Accessed December 19, 2025).

[11] Jia Y Lee S Kanda M Park P Edwards S Gao J . Sustainable age-friendly cities and communities in China: a scoping review and narrative assessment of national policies. Lancet Reg Health West Pac. (2025) 64:101723. doi: 10.1016/j.lanwpc.2025.101723 41281903 PMC12639885

[12] General Office of the Ministry of Housing and Urban-Rural Development of the People’s Republic of China Notice of the general Office of the Ministry of housing and urban-rural development on issuing the guidelines for Pocket Park construction (trial): Jianban Chenghan [2024] no. 214 (2024). Available online at: https://www.hunan.gov.cn/zqt/zcsd/202406/t20240624_33334517.html (Accessed December 21, 2025).

[13] Lu S Oh W Ooka R Wang L. Effects of environmental features in small public urban green spaces on older adults’ mental restoration: evidence from Tokyo. Int J Environ Res Public Health. (2022) 19:5477. doi: 10.3390/ijerph19095477, 35564870 PMC9100600

[14] Kaplan R Kaplan S. The Experience of Nature: A Psychological Perspective. Cambridge: Cambridge University Press (1989).

[15] Kaplan S. The restorative benefits of nature: toward an integrative framework. J Environ Psychol. (1995) 15:169–82.

[16] Ulrich RS. "Aesthetic and affective response to natural environment". In: Altman I Wohlwill JF, editors. Behavior and the Natural Environment. Boston, MA: Springer (1983). p. 85–125.

[17] Ulrich RS Simons RF Losito BD Fiorito E Miles MA Zelson M. Stress recovery during exposure to natural and urban environments. J Environ Psychol. (1991) 11:201–30.

[18] Ulrich RS. View through a window may influence recovery from surgery. Science. (1984) 224:420–1. 6143402 10.1126/science.6143402

[19] Hartig T Korpela K Evans GW Gärling T. A measure of restorative quality in environments. Scand Hous Plan Res. (1997) 14:175–94.

[20] Zhang Y Feng L Yan A. Exploring features of pocket parks that related to restorative effects: a systematic review. Urban Sci. (2025) 9:326. doi: 10.3390/urbansci9080326

[21] Peschardt KK Stigsdotter UK. Associations between park characteristics and perceived restorativeness of small public urban green spaces. Landsc Urban Plan. (2013) 112:26–39. doi: 10.1016/j.landurbplan.2012.12.013

[22] Xu J Qiu B Zhang F Zhang J. Restorative effects of pocket parks on mental fatigue among young adults: a comparative experimental study of three park types. Forests (Basel). (2024) 15:286. doi: 10.3390/f15020286

[23] Ohly H White MP Wheeler BW Bethel A Ukoumunne OC Nikolaou V . Attention restoration theory: a systematic review of the attention restoration potential of exposure to natural environments. J Toxicol Environ Health B Crit Rev. (2016) 19:305–43. doi: 10.1080/10937404.2016.1196155, 27668460

[24] Zhao J Abdul Aziz F Cheng Z Ujang N Zhang H Xu J . Post-occupancy evaluation of the improved old residential neighborhood satisfaction using principal component analysis: the case of Wuxi, China. ISPRS Int J Geo Inf. (2024) 13:318. doi: 10.3390/ijgi13090318

[25] Tan C Tang Y Wu X. Evaluation of the equity of urban park green space based on population data spatialization: a case study of a central area of Wuhan, China. Sensors (Basel). (2019) 19:2929. doi: 10.3390/s19132929, 31269765 PMC6651491

[26] Ren X Wang J Wang X Xue R Qi Y. Exploration of feasible measures for ageing-ready retrofitting of public space in older communities based on AHP-fuzzy comprehensive evaluation approach. Front Soc Sci Technol. (2024) 6:148–54. doi: 10.25236/FSST.2024.060125

[27] Cheng Q. Research on improving urban park green space landscape quality based on public psychological perception: a comprehensive AHP-TOPSIS-POE evaluation of typical parks in Jinan City. Front Psychol. (2025) 16:1418477. doi: 10.3389/fpsyg.2025.1418477, 39981382 PMC11839653

[28] Peng X Mohamed Afla MR. A multi-dimensional assessment of pocket park landscapes: insights from scenic beauty estimation and analytic hierarchy process in Dadukou District, Chongqing. Sustainability. (2025) 17:2020. doi: 10.3390/su17052020

[29] Huang J Song Y Sheng Y Zhang Y Hu D. Restorative potential assessment of public open space in old urban communities in the context of aging—a case study of Dabeizhuang Community in Maanshan, China. Buildings. (2024) 14:2671. doi: 10.3390/buildings14092671

[30] World Health Organization. World Report on Ageing and Health (2015). Available online at: https://www.who.int/publications/i/item/9789241565042 (Accessed December 25, 2025).

[31] Sugiyama T Ward Thompson C. Outdoor environments, activity and the well-being of older people: conceptualising environmental support. Environ Plan A. (2007) 39:1943–60. doi: 10.1068/a38226

[32] Korpela KM Ylén M Tyrväinen L Silvennoinen H. Determinants of restorative experiences in everyday favorite places. Health Place. (2008) 14:636–52. doi: 10.1016/j.healthplace.2007.10.008, 18037332

[33] Stevenson MP Schilhab T Bentsen P. Attention restoration theory II: a systematic review to clarify attention processes affected by exposure to natural environments. J Toxicol Environ Health B Crit Rev. (2018) 21:227–68. doi: 10.1080/10937404.2018.1505571, 30130463

[34] Golden BL Wasil EA Harker PT. The Analytic Hierarchy Process: Applications and Studies. Berlin, Heidelberg: Springer (1989).

[35] Zadeh LA. Fuzzy sets. Inf Control. (1965) 8:338–53.

[36] Ishikawa A Amagasa M Shiga T Tomizawa G Tatsuta R Mieno H. The max-min Delphi method and fuzzy Delphi method via fuzzy integration. Fuzzy Sets Syst. (1993) 55:241–53.

[37] Wang X Cao Y Zhong X Gao P. A new method of regional eco-environmental quality assessment and its application. J Environ Qual. (2012) 41:1393–401. doi: 10.2134/jeq2011.0390, 23099930

[38] Chiang Y-C Lei H-Y. Using expert decision-making to establish indicators of urban friendliness for walking environments: a multidisciplinary assessment. Int J Health Geogr. (2016) 15:40. doi: 10.1186/s12942-016-0071-7, 27846889 PMC5111263

[39] Li S-J Luo Y-F Liu Z-C Xiong L Zhu B-W. Exploring strategies for improving green open spaces in old downtown residential communities from the perspective of public health to enhance the health and well-being of the aged. J Healthc Eng. (2021) 2021:5547749. doi: 10.1155/2021/5547749, 35126893 PMC8814349

[40] Wei F Wang Y Chen L Liu Y. Renovation of informal green spaces in old urban residential communities in Chinese cities and related public perception investigation. Landsc Archit Front. (2020) 8:30–45. doi: 10.15302/J-LAF-0-020007

[41] Zhou Z Ye X Chen J Kang J. Effect of the visual landscape and soundscape factors on attention restoration in the public space of old residential areas by VR. Int J Acoust Vib. (2023) 28:300–9. doi: 10.20855/ijav.2023.28.31974

[42] Nordh H Østby K. Pocket parks for people—a study of park design and use. Urban For Urban Green. (2013) 12:12–7. doi: 10.1016/j.ufug.2012.11.003

[43] Peschardt KK Stigsdotter UK. Evidence for designing health promoting pocket parks. Archnet-IJAR. (2014) 8:149–64. doi: 10.26687/archnet-ijar.v8i3.341

[44] Peschardt KK Stigsdotter UK Schipperijn J. Identifying features of pocket parks that may be related to health promoting use. Landsc Res. (2016) 41:79–94. doi: 10.1080/01426397.2014.894006

[45] Zhang Y Hu Y Wei Y Xie Y. Can pocket parks be compared to community parks in the restoration effect of physical and mental health for young adults? A comparative experiment in high-density urban green spaces. Front Public Health. (2025) 13:1610497. doi: 10.3389/fpubh.2025.1610497, 40529708 PMC12171149

[46] Ma G Pellegrini P Ma J Shi L. Investigating the influence of elements in pocket parks on the psychological restoration of young people: a study from Guiyang and Chongqing in Southwest China. J Asian Archit Build Eng. (2025) 24:4420–32. doi: 10.1080/13467581.2024.2398204

[47] Maanshan Municipal Bureau of Statistics Urbanization level steadily improves and the degree of population ageing continues to deepen (2023). Available online at: https://tjj.mas.gov.cn/tjxx/2003870851.html (Accessed December 28, 2025).

[48] The Central People’s Government of the People’s Republic of China Notice on issuing the 14th five-year plan for healthy ageing (2022). Available online at: https://www.gov.cn/zhengce/zhengceku/2022-03/01/content_5676342.htm (Accessed December 29, 2025).

[49] World Health Organization. Global age-Friendly Cities: a guide. Geneva: World Health Organization (2007).

[50] Kou R Hunter RF Cleland C Ferguson S Schipperijn J Peng Q . Built environment influences on park visits for older adults: insights from a machine learning approach. Cities. (2025) 165:106143. doi: 10.1016/j.cities.2025.106143

[51] Zhang W Gao Y Li S Liu W Zeng C Gao L . Accessibility measurements for urban parks considering age-grouped walkers’ sectorial travel behavior and built environment. Urban For Urban Green. (2022) 76:127715. doi: 10.1016/j.ufug.2022.127715

[52] Balai Kerishnan P Maruthaveeran S. Factors contributing to the usage of pocket parks—a review of the evidence. Urban For Urban Green. (2021) 58:126985. doi: 10.1016/j.ufug.2021.126985

[53] Nordh H Hartig T Hagerhall CM Fry G. Components of small urban parks that predict the possibility for restoration. Urban For Urban Green. (2009) 8:225–35. doi: 10.1016/j.ufug.2009.06.003

[54] Fuller RA Irvine KN Devine-Wright P Warren PH Gaston KJ. Psychological benefits of greenspace increase with biodiversity. Biol Lett. (2007) 3:390–4. doi: 10.1098/rsbl.2007.0149, 17504734 PMC2390667

[55] Dallimer M Irvine KN Skinner AMJ Davies ZG Rouquette JR Maltby LL . Biodiversity and the feel-good factor: understanding associations between self-reported human well-being and species richness. Bioscience. (2012) 62:47–55. doi: 10.1525/bio.2012.62.1.9

[56] Zhao W Li H Zhu X Ge T. Effect of birdsong soundscape on perceived restorativeness in an urban park. Int J Environ Res Public Health. (2020) 17:5659. doi: 10.3390/ijerph17165659, 32764453 PMC7459586

[57] Payne SR. The production of a perceived restorativeness soundscape scale. Appl Acoust. (2013) 74:255–63. doi: 10.1016/j.apacoust.2011.11.005

[58] Zhang Z Wang Y Wu Z. Field study of thermal comfort of the elderly in tree-shaded areas of urban parks in the cold area of China. Sci Rep. (2025) 15:23276. doi: 10.1038/s41598-025-06785-1, 40604074 PMC12223223

[59] Li L Wang Z Zhen M Nan K. Study on outdoor thermal comfort of older people in age-friendly communities in cold regions. Energ Buildings. (2025) 329:115265. doi: 10.1016/j.enbuild.2024.115265

[60] Lin J Li X Lin J. Evaluation of age-appropriate public seats in comprehensive parks and sustainable design strategies based on the Kano-importance–performance analysis model. Sustainability. (2024) 16:6914. doi: 10.3390/su16166914

[61] Kimic K Polko P. The use of urban parks by older adults in the context of perceived security. Int J Environ Res Public Health. (2022) 19:4184. doi: 10.3390/ijerph19074184, 35409867 PMC8998194

[62] Lapham SC Cohen DA Han B Williamson S Evenson KR McKenzie TL . How important is perception of safety to park use? A four-city survey. Urban Stud. (2016) 53:2624–36. doi: 10.1177/0042098015592822, 34552299 PMC8455087

[63] Wu S Wu S Chen J Pan C. Predicting geriatric environmental safety perception assessment using LightGBM and SHAP framework. Sci Rep. (2025) 15:27444. doi: 10.1038/s41598-025-12541-2, 40721444 PMC12304219

[64] Weng Y Chen Q Lin X Chi Y Li K. Restorative effects of small urban parks: a multi-method study using eye-tracking and psychophysiological measures in Fuzhou, China. Front Public Health. (2025) 13:1667502. doi: 10.3389/fpubh.2025.1667502, 41368675 PMC12683676

[65] Murray TJ Pipino LL van Gigch JP. A pilot study of fuzzy set modification of Delphi. Hum Syst Manag. (1985) 5:76–80.

[66] Saaty TL. A scaling method for priorities in hierarchical structures. J Math Psychol. (1977) 15:234–81.

[67] Saaty TL. The Analytic Hierarchy Process: Planning, Priority Setting, Resource Allocation. New York: McGraw-Hill International Book Company (1980). p. 287.

[68] Saaty TL. How to make a decision: the analytic hierarchy process. Eur J Oper Res. (1990) 48:9–26. 10.1016/0377-2217(90)90060-o 11659401

[69] Aczél J Saaty TL. Procedures for synthesizing ratio judgements. J Math Psychol. (1983) 27:93–102.

[70] Triantaphyllou E Sánchez A. A sensitivity analysis approach for some deterministic multi-criteria decision-making methods. Decis Sci. (1997) 28:151–94.

[71] Friedman M. The use of ranks to avoid the assumption of normality implicit in the analysis of variance. J Am Stat Assoc. (1937) 32:675–701.

[72] Wilcoxon F. Individual comparisons by ranking methods. Biom Bull. (1945) 1:80–3.

[73] Holm S. A simple sequentially rejective multiple test procedure. Scand J Stat. (1979) 6:65–70.

[74] Forman E Peniwati K. Aggregating individual judgments and priorities with the analytic hierarchy process. Eur J Oper Res. (1998) 108:165–9.

[75] Escobar MT Aguarón J Moreno-Jiménez JM. A note on AHP group consistency for the row geometric mean priorization procedure. Eur J Oper Res. (2004) 153:318–22. doi: 10.1016/S0377-2217(03)00154-1

[76] Luo S Xie J Furuya K. Assessing the preference and restorative potential of urban park blue space. Land. (2021) 10:1233. doi: 10.3390/land10111233

[77] Saelens BE Frank LD Auffrey C Whitaker RC Burdette HL Colabianchi N. Measuring physical environments of parks and playgrounds: EAPRS instrument development and inter-rater reliability. J Phys Act Health. (2006) 3:S190–207. doi: 10.1123/jpah.3.s1.s190, 28834520

[78] Duan Y Bai H Yang L Li S Zhu Q. Impact of seasonal changes in urban green spaces with diverse vegetation structures on college students' physical and mental health. Sci Rep. (2024) 14:16277. doi: 10.1038/s41598-024-67075-w, 39009702 PMC11251041

[79] Herzog TR Maguire CP Nebel MB. Assessing the restorative components of environments. J Environ Psychol. (2003) 23:159–70. doi: 10.1016/S0272-4944(02)00113-5

[80] Zhan P Guo Q Chen H Wu Y. Antecedents and consequences of park crowding: linking park attractiveness, perceived crowding, and revisit intention. Landsc Urban Plan. (2024) 245:105015. doi: 10.1016/j.landurbplan.2024.105015

