# Supplementary material for: Assessing the restorative environment of pocket parks in old urban districts of Chinese cities in the context of healthy aging
Source: Front Public Health. 2026 Jul 15;14:1861979. doi: 10.3389/fpubh.2026.1861979 (PMC13418448; doi:10.3389/fpubh.2026.1861979)
Supplement: Supplementary file 4 [file Table_4.DOCX]

Basic information on the expert panel

| No. | Area of expertise | Type of institution | Work profile |
| --- | --- | --- | --- |
| 1 | Landscape planning and urban regeneration | Higher education system | Teaching and research; long-term engagement in research on urban public space and community regeneration |
| 2 | Environmental design and age-friendly design | Higher education system | Teaching and research; participated in studies on age-friendly spaces in the past three years |
| 3 | Urban design and public-space evaluation | Higher education system | Teaching and research; engaged in research on urban open spaces and healthy human settlements |
| 4 | Landscape architecture and community green-space planning | Higher education system | Teaching and research; participated in studies on community green-space and pocket park renewal |
| 5 | Geriatric medicine and health promotion | Medical system | Clinical practice and health management; familiar with older adults’ daily activities and health needs |
| 6 | Rehabilitation medicine and geriatric health | Medical system | Clinical practice and rehabilitation services; participated in health service work for older adults |
| 7 | Public health and healthy cities | Research institution | Research; focusing on environmental exposure, health behavior, and health in later life |
| 8 | Community planning and urban regeneration | Design industry | Planning and design practice; participated in old community renewal and public-space improvement |
| 9 | Landscape design and pocket park practice | Design industry | Design practice; participated in urban micro-green space and pocket park projects |
| 10 | Age-friendly environmental design | Design industry | Design practice; participated in elderly community renewal and public-space design |
| 11 | Architectural design and healthy human settlements | Design industry | Design practice; focusing on residential environmental quality and age-friendly space creation |
| 12 | Community governance and public services | Elderly care institution | Community services; familiar with older residents’ daily activities and spatial use needs |
| 13 | Urban green-space systems and ecological planning | Higher education system | Research and planning practice; participated in urban green-space and open-space evaluation |
| 14 | Environmental psychology and post-occupancy evaluation | Higher education system | Teaching and research; engaged in studies on spatial perception, post-occupancy evaluation, and healthy environments |

Demographic and Park-Use Characteristics of Older Respondents

| Variable | Category | Total sample n (%) | Zhenqu n (%) | Kangle n (%) | Zhumeng n (%) |
| --- | --- | --- | --- | --- | --- |
| Sample size |  | 278 (100.0) | 94 (100.0) | 91 (100.0) | 93 (100.0) |
| Gender | Male | 126 (45.3) | 43 (45.7) | 41 (45.1) | 42 (45.2) |
|  | Female | 152 (54.7) | 51 (54.3) | 50 (54.9) | 51 (54.8) |
| Age | 60–69 years | 159 (57.2) | 54 (57.4) | 53 (58.2) | 52 (55.9) |
|  | 70–79 years | 109 (39.2) | 36 (38.3) | 35 (38.5) | 38 (40.9) |
|  | ≥80 years | 10 (3.6) | 4 (4.3) | 3 (3.3) | 3 (3.2) |
| Travel time to park | ≤10 min | 181 (65.1) | 60 (63.8) | 61 (67.0) | 60 (64.5) |
|  | 11–20 min | 76 (27.3) | 26 (27.7) | 23 (25.3) | 27 (29.0) |
|  | >20 min | 21 (7.6) | 8 (8.5) | 7 (7.7) | 6 (6.5) |
| Visit frequency | Almost every day | 164 (59.0) | 56 (59.6) | 55 (60.4) | 53 (57.0) |
|  | Several times per week | 87 (31.3) | 29 (30.9) | 28 (30.8) | 30 (32.3) |
|  | Occasional visits | 27 (9.7) | 9 (9.6) | 8 (8.8) | 10 (10.8) |
| Duration of each stay | ≤30 min | 68 (24.5) | 21 (22.3) | 23 (25.3) | 24 (25.8) |
|  | 31–60 min | 142 (51.1) | 48 (51.1) | 47 (51.6) | 47 (50.5) |
|  | >60 min | 68 (24.5) | 25 (26.6) | 21 (23.1) | 22 (23.7) |
| Main activities  (Multiple choice) | Walking | 206 (74.1) | 68 (72.3) | 69 (75.8) | 69 (74.2) |
|  | Resting | 158 (56.8) | 55 (58.5) | 50 (54.9) | 53 (57.0) |
|  | Fitness exercise | 132 (47.5) | 48 (51.1) | 40 (44.0) | 44 (47.3) |
|  | Social chatting | 121 (43.5) | 42 (44.7) | 39 (42.9) | 40 (43.0) |
|  | Looking after children | 49 (17.6) | 16 (17.0) | 17 (18.7) | 16 (17.2) |
|  | Other | 18 (6.5) | 6 (6.4) | 5 (5.5) | 7 (7.5) |
